# Body composition variables as predictors of NAFLD by ultrasound in obese children and adolescents

**DOI:** 10.1186/1471-2431-14-25

**Published:** 2014-01-29

**Authors:** Paula Alves Monteiro, Barbara de Moura Mello Antunes, Loreana Sanches Silveira, Diego Giulliano Destro Christofaro, Rômulo Araújo Fernandes, Ismael Forte Freitas

**Affiliations:** 1 Department of Physical Education, University Estadual Paulista, Campus of Rio Claro, São Paulo, Brazil; 2 Department of Physiotherapy, University Estadual Paulista, Campus of Presidente Prudente, São Paulo, Brazil; 3 Department of Physical Education, University Estadual Paulista, Campus of Presidente Prudente, São Paulo, Brazil; 4 Universidade Estadual Paulista “Júlio de Mesquita Filho”, 305, Roberto Simonsen St. Presidente Prudente, São Paulo 19060-900, Brazil

**Keywords:** Body composition, Obesity, Fatty liver, Children, Adolescents

## Abstract

**Background:**

Nonalcoholic fatty liver disease (NAFLD) is a disorder associated with excessive fat accumulation, mainly in the intra-abdominal region. A simple technique to estimate abdominal fat in this region could be useful to assess the presence of NAFLD, in obese subjects who are more vulnerable to this disease. The aim of this cross-sectional study was to verify the reliability of waist circumference and body composition variables to identify the occurrence of NAFLD in obese children and adolescents.

**Methods:**

Sample was composed of 145 subjects, aged 11 to 17 years. Assessments of waist circumference (WC), trunk fat mass (TFM) and fat mass (FM) by dual-energy X-ray absorptiometry (DXA) and ultrasound for diagnosis of NAFLD and intra-abdominal adipose tissue (IAAT) were used. Correlation between variables was made by Spearman’s coefficients; ROC curve parameters (sensitivity, specificity, area under curve) were used to assess the reliability of body composition variables to assess the presence of NAFLD. Statistical significance was set at 5%.

**Results:**

Significant correlations were observed between NAFLD and WC (p = 0.001), TFM (p = 0.002) and IAAT (p = 0.001). The higher values of area under the ROC curve were for WC (AUC = 0.720), TFM (AUC = 0.661) and IAAT (AUC = 0.741).

**Conclusions:**

Our findings indicated that TFM, IAAT and WC present high potential to identify NAFLD in obese children and adolescents.

## Background

Obesity is considered a multifactorial disease and, usually, results from positive energy balance, influenced by endogenous and exogenous factors [1]. Several metabolic disorders are associated with obesity, such as nonalcoholic fat liver disease (NAFLD) characterized by accumulation of fat in the hepatocyte [2].

Subjects with high amount of abdominal fat present the lipolytic activity of adipocyte more activated, leading to a higher liberation of free fatty acids [3,4] in the portal venous system, and, as result, the liver is more exposed to a high amount of fat which can increase the risk of NAFLD in five to six times [5].

The use of appropriate methods to estimate body fat and diagnose NAFLD is extremely important [6]. The NAFLD diagnosis may be made by several methods, such as liver biopsy and liver enzymes function and ultrasound as an imaging technique [7].

An ultrasound of the abdominal region is a practical, reliable and economic technique to diagnose NAFLD [8], and, additionally, allows the measurement of intra-abdominal fat thickness [9]. Besides, the central adiposity can be estimatedby other methods, such as the dual-energy X-ray absorptiometry (DEXA) [10] which presents high correlation with intra-abdominal adipose tissue (IAAT) and can be used as indicator of metabolic diseases, including insulin resistance and dyslipidemia, and, consequently, NAFLD [11,12].

According to Koning et al. [13]. some anthropometric measurements, including abdominal and waist circumferences, can contribute to estimate IAAT, and be useful in the diagnosis of NAFLD, with some advantages such as easy applicability, low cost and the nonrequirement of specialized training.

Thus, the aim of the present study was to verify the reliability of waist circumference and body composition variables to identify the occurrence of NAFLD in obese children and adolescents.

## Methods

### Participants and setting

This crossectional study was developed in the city of Presidente Prudente, located in the state of São Paulo, Brazil. The participants were invited, through media advertisement (newspaper, television and internet). The inclusion criteria were: (i) Be obese, classified according to the recommendations published by Cole et al. [14], (ii) Aged between 11 and 17 years at the time of initial evaluation, (iii) Do not present any clinical problem that influence physical activity practice, and (iv) Informed consent form signed by the parents and/or guardians. A total of 145 subjects met these criteria and composed the sample. This research was approved by the Ethics Committee of FCT/UNESP (Protocol number: 07/2009).

### Anthropometry

Body mass was measured with a Filizola electronic scale electronic scale (precision 0.1 kg) (Filizzola PL 150, Filizzola Ltda) and the height with a wall-mounted stadiometer [precision 0.1 cm (Sanny®, São Paulo, Brazil)]. The waist circumference (WC) was measured at lowest circumference between the superior border of the iliac crest and below the lowest rib with a inelastic tape [precision 0.1 cm (Sanny®, São Paulo, Brazil)], with the subjects in standing position, breathing normally and with arms relaxed beside the trunk. The record was made at the end of a normal expiration.The All anthropometric measurements were made following the recommendations proposed by Lohman et al. [15]. The calculation of body mass index (BMI) was performed by the equation: body mass (Kg)/height^2^ (m) [16].

### Dual energy X-Ray absorptiometry

Body composition was estimated by a Dual-energy X-ray absorptiometry (DEXA) scanner (Lunar DPX-NT; General Electric Healthcare, Little Chalfont, Buckinghamshire), with software version 4.7. The method estimated the body composition by fractionating the body into three anatomical compartments: fat-free mass (FFM), fat mass (FM) and bone mineral content. The assessment was carried out in approximately 15 minutes, and the subjects remained still and in a supine position throughout the scan, wearing light clothes. The results of fat-free mass (FFM), fat mass (FM) and trunk fat mass (TFM) were expressed in kilograms and percentage. All DEXA measurements were carried out at the University laboratory in a controlled temperature room. The DEXA equipment was calibrated each morning, before the beginning of the measurements, by the same researcher, according to the references provided by the manufacturer.

### Nonalcoholic fatty liver disease

The ultrasound examination of the upper abdomen was used to identify the presence of NAFLD. The diagnostic criteria were: (i) Absence: normal echogenicity and (ii) Presence: alteration of the fine echoes, visualization of diaphragm and intra hepatic vessel borders according to Saadeh et al [17]. All examinations were performed by the same qualified radiologist, using a *TOSHIBA Eccocee* having a convex transducer of 3.7 Mhz. All subjects followed the recommendation of fastting for 4 hours prior to evaluation according to medical literature.

### Intra-abdominal adipose tissue

The IAAT was measured by ultrasound examination, using a TOSHIBA Eccocee, with convex transducer of 3.7 Mhz 1 cm above the umbilical scar. The IAAT was defined as the distance between the skin and external face of the rectus abdominal muscle, and visceral fat was defined as the distance between the internal face of the same muscle and the anterior wall of the aorta previously described by Ribeiro-Filho et al. [18].

### Statistical analysis

The Kolmogorov-Smirnov test was used to verify the distribution of variables. The non-parametric descriptive statistics for numeric variables were expressed as median and interquartile range (IQR). Spearman’s correlation coefficients were used to assess potential relationship between variables, and the ROC curve parameters (sensitivity, specificity, area under curve [AUC] predictive positive value [PPV] and predictive negative value [PNV]) were used to verify the characteristics of the independent variables. All analyses were performed using BioEstat software (release version 5.0) and the statistical significance was set at p-value <5%.

## Results

The general characteristics of subjects are described by gender in Table 1. Weight, height, BMI, WC, FM and IAAT presented significant differences between genders. The prevalence of NAFLD was 31%, and in the male group was statistically higher than in female.

**Table 1:** General characteristics of obese children and adolescents, according to gender

	**Male**	**Female**	***p*-value**
	**Median (IQR)**	**Median (IQR)**	
**Age (years)**	13.0 (6.0)	13.0(5.0)	0.888
**Weight (kg)**	84.4(80.6)	73.5(66.8)	0.001
**Height (cm)**	163.5(38.3)	159.7(35.3)	0.001
**BMI (kg/cm**^**2**^**)**	31.2(19.8)	29.2(18.4)	0.002
**WC (cm)**	95.1(42.0)	85.5(45.0)	0.001
**FM (kg)**	36.8(44.9)	33.6(39.1)	0.035
**TFM (kg)**	17.2(21.1)	15.9(23.3)	0.055
**IAAT (cm)**	4.5(9.4)	3.3(7.8)	0.001
**Categorical variable (n [%])**			
**NAFLD**	30 (40%)	15 (21.4%)	0.016

IQR = interquartile range; BMI = body mass index; WC = waist circumference; FM = fat mass; TFM = trunk fat mass; IAAT = intra-abdominal adipose tissue; NAFLD = non-alcoholic fat liver disease; NS = No significant.

Table 2 shows the Spearman’s correlation coefficient where significant relationship between NAFLD and IAAT, WC and TFM were observed.

**Table 2:** **Spearman correlation (*r *) between NAFLD, anthropometric and body composition variables in obese children and adolescents (n = 145)**

	**Non-alcoholic fat liver disease**	
**Variables**	***r***	***p*-value**
**Overall**		
**WC (cm)**	0.352	0.001
**TFM (kg)**	0.259	0.002
**IAAT (cm)**	0.387	0.001
**Male**		
**WC (cm)**	0.136	0.244
**TFM (kg)**	0.128	0.271
**IAAT (cm)**	0.340	0.003
**Female**		
**WC (cm)**	0.451	0.001
**TFM (kg)**	0.391	0.001
**IAAT (cm)**	0.383	0.001

NAFLD = nonalcoholic fatty liver disease; WC = waist circumference; TFM = trunk fat mass; FM = fat mass; IAAT = intra-abdominal adipose tissue.

The AUC values ranged from 0.661 to 0.741 (WC = 0.720 [AUC_95%CI_ = 0.636-0.804]; IAAT = 0.741 [AUC_95%CI_ = 0.659-0.824]; TFM = 0.661 [AUC_95%CI_ = 0.565-0.757]), and the comparison between WC and IAAT (difference between AUC = 0.023; *p*-value = 0.701), WC and TFM (difference between AUC = 0.057; *p*-value = 0.097) and TFM and IAAT (difference between AUC = 0.080; *p*-value = 0.227), did not show statistical differences.

TheIAAF was used as reference, and the analysis of sensitivity and specificity showed that TFM presented higher specificity and WC higher sensitivity. PPV, and PNV of TFM and WC were similar (Table 3).

**Table 3:** Sensitivity, specificity and accuracy of body composition variables to diagnostic NAFLD in obese individuals

**Variables**	**Sensitivity**	**Specificity**	**PPV**	**PNV**
**Overall**				
**WC**	0.667	0.640	45.4	81.0
**TFM**	0.733	0.540	41.8	81.8
**IAAT**	0.756	0.610	46.5	84.8
**Male**				
**WC**	0.567	0.600	51.6	56.1
**TFM**	0.533	0.537	42.1	44.3
**IAAT**	0.600	0.600	46.1	48.2
**Female**				
**WC**	0.733	0.704	45.2	57.4
**TFM**	0.533	0.611	26.7	43.1
**IAAT**	0.400	0.833	40.1	83.3

PPV = predictive positive value; PNV = predictive negative value; WC = waist circumference; TFM = trunk fat mass; IAAT = intra-abdominal adipose tissue; NAFLD = non-alcoholic fat liver disease; IAAT = intra-abdominal adipose tissue.

## Discussion

The aim of the present study was to verify the reliability of anthropometric and body composition variables thatcould be used to identify the occurrence of NAFLD in obese children and adolescents. The prevalence of NAFLD was 31% for all samples. Male presentedhigher prevalence (40%) than girls (28.0%). Similar results were observed by Nadeau et al. [19] that found high prevalence of NAFLD in adolescents (74%) and reported that the NAFLD is more common in male and Hispanic subjects. Denzer et al. [20] also found similar prevalence in boys (41.1%) and girls (17.2%) aged 8 to 19 years.

The presence of NAFLD plays an important role in the development of other unhealthy outcomes. Subjects with high amounts of fat in the liver are more vulnerable to negative effects of oxygen reactive species [21]. Schwimmer at al. [22] showed that overweight children with NAFLD present higher fasting glucose, insulin, total cholesterol, LDL-cholesterol, triglycerides and high blood pressure than those without NAFLD. Moreover, NAFLD is strongly associated with metabolic syndrome in pediatric populations [23] and is considered the hepatic manifestation of this syndrome in adults [24].

Excess of body fat, mainly abdominal fat [25], is related to NAFLD and IAAT is considered a determinant factor to increase prevalence and good predictor to identify the risk for development of NAFLD [26]. Our studies showed significant correlation between all independent variables and the presence of the NAFLD. Previous studies have reported similar findings for WC [26,27] and, according to Lin et al. [28], the measurement of WC is better than BMI to predict liver steatosis and is considered as a substitute of central obesity assessment.

Our findings also indicated that IAAT and WC were similar predictors of NAFLD and these two measurements are correlated between them [29]. Therefore, the positive relationships between WC with IAAT and WC with NAFLD indicate that WC is a proxy of the abdominal obesity and, there is a plausible support for the use of this anthropometric measure as indicator of NAFLD in obese pediatric populations.

According to the results of ROC curve, WC and IAAT were the two variables with highest AUC. There were moderate values for sensitivity (ability of WC to identify NAFLD) and specificity (ability of WC to diagnose the absence NAFLD) of adolescents. PPV and PNV support our hypothesis that WC is a more specific than sensitive index. In a previous epidemiologic study with Korean adults aged 20 to 88 years, the authors compared the usefulness of obesity indices, measured by computed tomography, DEXA and WC to identify NAFLD. They concluded that WC was a good predictor of IAATand usefull for diagnosing NAFLD [12]. Our results indicate similar findings, and suggest the use of WC measurement, in school settings, to identify children and adolescents at risk of NAFLD.

Previous studies presented WC cutoff for adults (89 cm for men and 84 cm for women) to indicate higher risk of NAFLD [12], however, for children and adolescents only one study was found in the literature that provides cutoff for WC which use percentile values as a tool to assess the impact of abdominal adipose tissue as risk factor for chronic diseases in terms of public health, but this study did not refer that it can these cut-off can be applied to assess the risk to develop NAFLD [30].

One of the limitations of the present study is the use of only one diagnostic method of NAFLD, thus the double-diagnostic would enrich our results [31].

## Conclusions

We concluded that body composition variables measured by anthropometry and DEXA, may be used as indicators of NAFLD in children and adolescents. Our findings point out that WC could be an interesting tool to identify children and adolescents at increased risk of NAFLD, but further efforts should be focused in the development of age-adjusted cutoffs for these populations.

## Abbreviations

NAFLD: Nonalcoholic fat liver disease; DEXA: Dual-energy X-ray absorptiometry; IAAT: Intra-abdominal adipose tissue; FFM: Fat-free mass; FM: Fat mass; WC: Waist circumference; BMI: Body mass index; TFM: Trunk fat mass; IQR: Interquartile range; AUC: Area under curve; PPV: Predictive positive value; PNV: Predictive negative value.

## Competing interests

The authors declare that they have no competing of interests.

## Authors’ contributions

PAM participated in the design of the study, was the main responsible for collection, analysis and interpretation of data, and also drafting the manuscript; BMMA carried out the Dual energy X-ray absorptiometry involved in analysis and interpretation of data and drafted the manuscript. LSS carried out the immunoassays and also in critical revision of the paper; carried out the immunoassays and also in critical revision of the paper; DGDC participated in the design of the study and reviewed the manuscript. RAF participated in the design of the study and performed the statistical analysis. IFFJ conceived the study and critically revised the manuscript. All authors read and approved the final manuscript**.**

## Pre-publication history

The pre-publication history for this paper can be accessed here:

http://www.biomedcentral.com/1471-2431/14/25/prepub
